# Extraction and Sensitive Detection of Toxins A and B from the Human Pathogen *Clostridium difficile* in 40 Seconds Using Microwave-Accelerated Metal-Enhanced Fluorescence

**DOI:** 10.1371/journal.pone.0104334

**Published:** 2014-08-27

**Authors:** Lovleen Tina Joshi, Buddha L. Mali, Chris D. Geddes, Les Baillie

**Affiliations:** 1 Cardiff School of Pharmacy and Pharmaceutical Sciences, Cardiff University, Cardiff, United Kingdom; 2 Institute of Fluorescence, University of Maryland, Baltimore County, Baltimore, Maryland, United States of America; Charité, Campus Benjamin Franklin, Germany

## Abstract

*Clostridium difficile* is the primary cause of antibiotic associated diarrhea in humans and is a significant cause of morbidity and mortality. Thus the rapid and accurate identification of this pathogen in clinical samples, such as feces, is a key step in reducing the devastating impact of this disease. The bacterium produces two toxins, A and B, which are thought to be responsible for the majority of the pathology associated with the disease, although the relative contribution of each is currently a subject of debate. For this reason we have developed a rapid detection assay based on microwave-accelerated metal-enhanced fluorescence which is capable of detecting the presence of 10 bacteria in unprocessed human feces within 40 seconds. These promising results suggest that this prototype biosensor has the potential to be developed into a rapid, point of care, real time diagnostic assay for *C. difficile*.

## Introduction

*Clostridium difficile* is a spore forming, toxin-producing bacterium which is currently the principal cause of healthcare associated diarrhea in the western world. In the USA in 2007 there were 284 875 infections while in the UK during the same period the pathogen was linked to the death of 8 324 individuals [1], [2]. The bacterium currently presents a considerable challenge to healthcare professionals and has stimulated researchers to develop improved diagnostics and medical countermeasures.

In healthy individuals the spore form of the bacterium is carried in the gut with no ill effects. Subsequent disruption of the gut flora, usually by broad spectrum antibiotics, enables the bacterium to proliferate and release two toxins, a 308 kDa enterotoxin (toxin A) and a 270 kDa cytotoxin (toxin B) which are responsible for symptoms ranging from mild diarrhea to fatal colitis [3].

The genes encoding the production of these toxins, *tcdA* and *tcdB*, are located within a 19.6 kb pathogenicity locus (PaLoc) which is present in all toxin producing strains [4]–[6]. The relative contribution of each toxin to pathogenicity is currently a subject of debate and thus the ability to detect the presence of both would greatly enhance diagnostic capability [7], [8].

The current gold standard for *C. difficile* diagnosis is the cell cytotoxin neutralization assay (CCTA) which can only detect toxin B, has 99–100% specificity and 85–100% sensitivity, and takes between 48–72 hours to generate a result [3], [9]. To reduce the time for detection, other methods have been developed such as enzyme immunoassays (EIAs) which detect toxins A and B, and glutamate dehydrogenase [9], [10]. These assays are easy to perform and give rapid results (15–20 minutes); however, they currently suffer from low sensitivity [11]–[14].

DNA methods based on the Real Time Polymerase Chain Reaction (RT PCR) have been developed to detect the genes associated with toxin production and the current commercially available systems, the ProGastro assay (Cepheid Smart Cycler), GeneXpert (Cepheid), and the BD Gene Ohm assay, all target toxin B [15], [16]. Although these assays are highly sensitive they are labor intensive and costly due to the need to include a purification step to remove inhibitory biological materials [11], [16]–[18].

A new platform technology called microwave-accelerated metal-enhanced fluorescence (MAMEF) has recently been developed which eliminates the need for extensive sample purification. The approach has been successfully employed to detect a range of human pathogens including *Bacillus anthracis, Salmonella typhimurium* and *Chlamydia trachomatis* [19]–[22]. The approach combines two technologies, metal enhanced fluorescence (MEF) to optically amplify fluorescence signatures and low power microwave heating to accelerate the reaction kinetics of the assay. Combined together they yield a system with a level of sensitivity comparable to RT PCR, but with a much shorter turnaround time of ∼60 seconds, and at a significantly lower cost.

The underlying principal of the technology is based on the selective heating of water, whereby the aqueous medium is heated via microwave power. As the metal is not heated, a temperature gradient between the cold metal surface and the warm aqueous solution is formed, which facilitates mass transport to the surface and allows DNA, or other biomarkers, to be recognized when the assay is complete and the fluorophore is in close proximity to the metal [22]. Non-radiative energy transfer occurs between fluorophores and plasmon electrons in a non-continuous film. The metal surface itself radiates the coupled fluorescence quanta [23]. As a consequence the assay does not require the prior removal of organic material such as blood and feces and is thus able to directly detect the presence of a bacterial target in clinical samples.

A system capable of detecting the presence of *C. difficile* spores in patient feces within 5 minutes would represent a considerable improvement over current capabilities. Such a system could potentially impact on the quality of clinical care as early treatment would limit disease progression and reduce cross transmission to other patients.

In this paper we describe our efforts to develop a MAMEF based system capable of detecting *C. difficile* spores in human feces. Using DNA probes specific to toxin A and B we were able to detect a few as 10 spores in 500 µl of unprocessed human feces within 40 seconds, suggesting that this approach has the potential to be developed into a rapid point of care, real time diagnostic assay.

## Materials and Methods

### Bacterial Strains, growth conditions and genomic DNA

*C. difficile* strain CD630 was obtained for culture from the NCTC, Public Health England, UK. BHI agar and broth (Oxoid; Remel Inc, KS, USA) supplemented with 0.1% of the bile salt sodium taurocholate (Sigma Aldrich, USA) was used for routine culture of *C. difficile* at 37°C in a 3.4 L anaerobic jar (Oxoid, UK) with an anaerobic gas generating kit (Oxoid, UK). Phosphate buffered saline (PBS; Sigma Aldrich, USA) constituting 0.01 M phosphate buffer, 0.0027 M potassium chloride and 0.137 M sodium chloride was made by dissolving 1 tablet in 200 ml diH_2_O. Human feces was provided by a healthy volunteer. Genomic DNA (gDNA) from *C. difficile* strain CD630 (5 µg) was obtained from ATCC, USA as a control.

### Genomic DNA Extraction from *C. difficile* using Chelex 100

Genomic DNA was extracted from *C. difficile* and other species (Table S1; Table S2) as described previously [24].

### *C. difficile* toxin A and B Probe Design

Probes were designed to recognize nucleotide sequences within conserved regions of toxins A and B. As part of the design process we incorporated features which would enable us to utilize the probes in a future MAMEF assay. The anchor probe for the *C. difficile* assay was designed to be 17 nucleotides in length and to be separated from the 22 nucleotide fluorescent detector probe by a stretch of 5 nucleotides. The anchor probe binds target DNA while the detector probe subsequently binds at a distance which positions the fluorophore optimally for biomolecular recognition to occur [22]. The use of anchor and detector probes also allows for two levels of sensitivity within the assay for each toxin (Table 1).

**Table 1:** Table of Oligonucleotides.

	Toxin A	Toxin B
Anchor Probe	Thiol-TTTTT-TTTAATACTAACACTGC	Thiol-TTTTTT-CAAGACTCTATTATAG
Capture Probe	Alexa-488-TGTTGCAGTTACTGGATGGCAA	Alexa-594-TAAGTGCAAATCAATATGAAG
Synthetic oligonucleotides	AAATTATGATTGTGACGTAATCCCAATACAACGTCAATGACCTACCGTT	AGTTCTGAGATAATATCTAATCCCAATATTCACGTTTAGTTATACTTG

Anchor and capture probes, and synthetic oligonucleotides target regions used in the MAMEF assay for toxins A and B are shown, along with the corresponding fluorophore used.

Designed probes were tested against a representative collection of 58 *C. difficile* isolates; including blood culture and variant strains (only produced toxin B; ribotypes 017, 047, 110), obtained from the National Anaerobic Reference Unit, Cardiff, Wales, courtesy of Dr. Val Hall.

### Preparation of DIG-labeled DNA Hybridization Probes

PCR was performed using *Taq* DNA polymerase core kit (Qiagen, Crawley, United Kingdom). Hybridization probes were labeled with the DIG (Digoxigenin) PCR dNTP labeling mix (Roche Diagnostics, UK) in 20 µl reactions as described previously [25]. PCR conditions are as described previously (Figure S3; Table S2) [25]. PCR products were analyzed via gel electrophoresis on a 1% agarose gel at 85 V (Biorad Mini sub cell GT, UK).

### Dot blot hybridization and macro-arraying gDNA onto a positively charged nylon membrane

For dot blot hybridization, gDNA was macroarrayed onto a positively charged nylon membrane using dot blot apparatus (Figure S3; Flexys robotic workstation, Genomic Solutions Ltd, UK). Dot blot hybridization was performed as described previously [26], [27].

### Dot blots of Human Metagenomic gut DNA & Ethics Statements

The human gut environment contains approximately 10^12^/g bacteria [28]. Therefore the probes designed to detect *C. difficile* must be able to specifically detect the toxin genes amongst the numerous bacteria present. To further confirm the specificity of the designed probes, metagenomic DNA samples from ten human volunteers were obtained from Cardiff School of Biosciences, Wales, Cardiff, UK, courtesy of Dr. Julian Marchesi. The metagenome was extracted from feces from humans in Zanzibar, Cote d'Ivoire and the UK [29], [30]. Original sample collection in the UK was approved by Cardiff University Biosciences Local Ethics Committee as healthy samples with informed consent. Original sample collection from Cote d'Ivoire was approved by the ethical review boards of the ETH Zurich, Switzerland (2006–23), the University of Basel (EKBB), Switzerland (224/06), and the Ministry of Health in Côte d'Ivoire (5782/MSHP/CAB/CNESVS/06) [29]. The original sample collection from Zanzibar was approved by the institutional research commission of the Swiss Tropical and Public Health Institute (Basel, Switzerland). Ethical clearance was obtained from the Ethics Committee of the Ministry of Health and Social Welfare (MoHSW) in Zanzibar (application number 16) [30].

### Deposition of gold triangles onto glass substrates to microwave and lyse *C. difficile*

**Figure 1:**
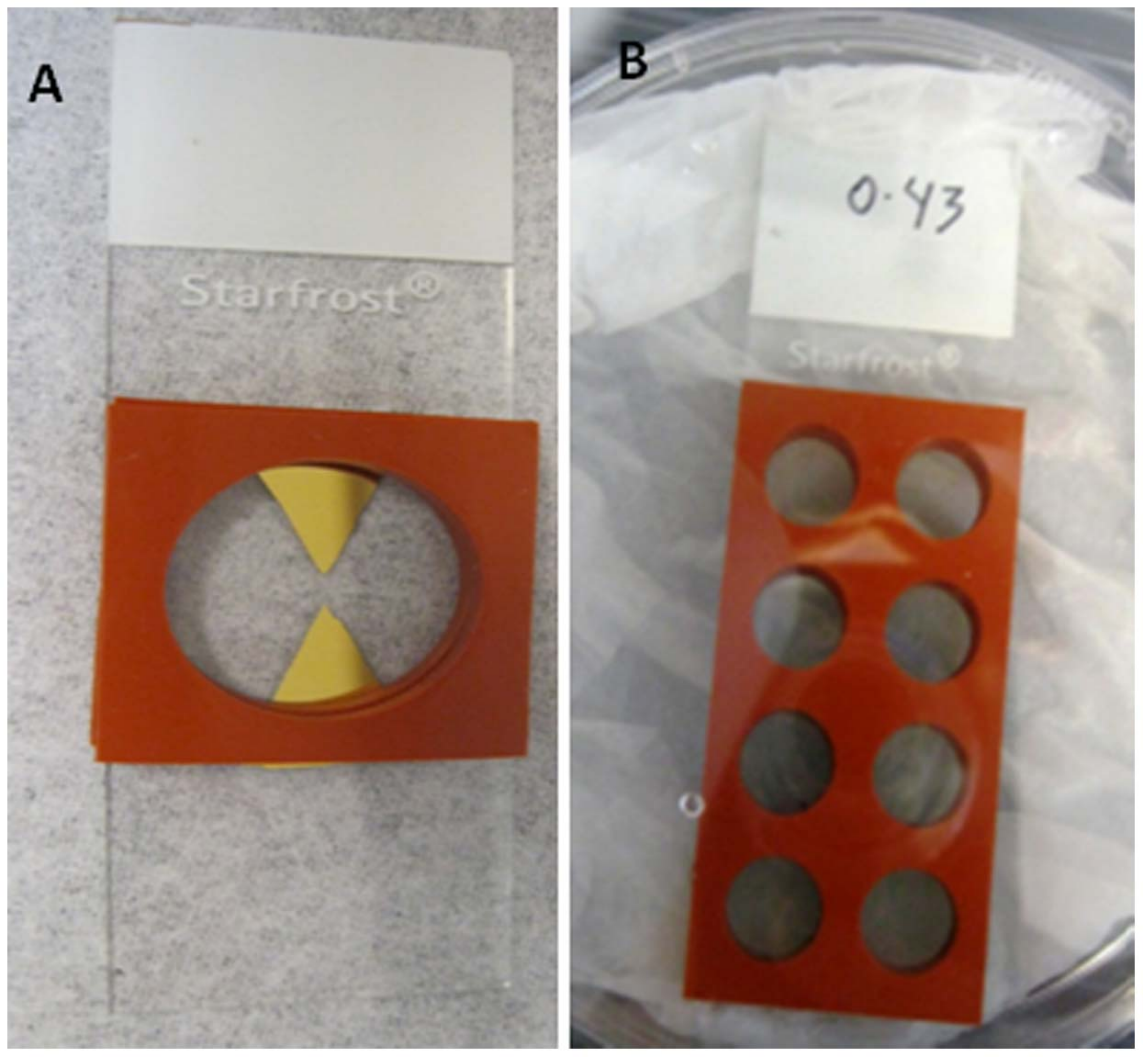
Gold lysing triangle on Starfrost slide and a typical Silver Island Film (SiFs). (A) Gold triangle with silicone isolators added to create lysing chamber. (B) The SiF here is at an OD_450_ of 0.43 and has multi-well silicone isolators added. Each well can hold an individual DNA assay reaction; hence multiple repeats can be conducted.

To focus the microwave power in the microwave, gold lysing triangles were used, provided by the Institute of Fluorescence (Figure 1). Deposition and preparation as described previously [22].

### The release of *C. difficile* DNA from spores using microwave irradiation

*C. difficile* (1×10^5^ cfu/ml) was suspended into PBS buffer and 500 µl added into the gold tie lysing chamber and exposed to a 15 s microwave pulse at 80% power in a GE microwave Model No. JE2160BF01, kW 1.65 (M/W). The microwaved solution was examined for the presence of viable organisms. Further experiments involved spiking human feces diluted with PBS buffer with varying concentrations of *C. difficile* and then irradiating. Each experiment was conducted in triplicate.

### Bacterial quantification after focused microwave irradiation

Control samples (1.33×10^7^ cfu/ml) were drop counted as described previously [31], [32]. Before DNA release, samples were heated at 80°C for 10 min to ensure removal of any vegetative cells. After lysis the numbers of remaining viable spores (cfu/ml) were enumerated.

### Gel electrophoresis of samples

Post microwave irradiation the sample was centrifuged at 3000 g for 15 min (Heraeus Primo R; Fisher Scientific, USA) and the supernatant resuspended in 1∶2 ethanol. This supernatant was resuspended in 100 µl sterile deionized water. Samples were analyzed via gel electrophoresis on a 2% agarose gel at 70 V (Biorad Mini sub cell GT, UK).

### Formation of Silver Island Films (SiFs) on glass substrates

**Figure 2:**
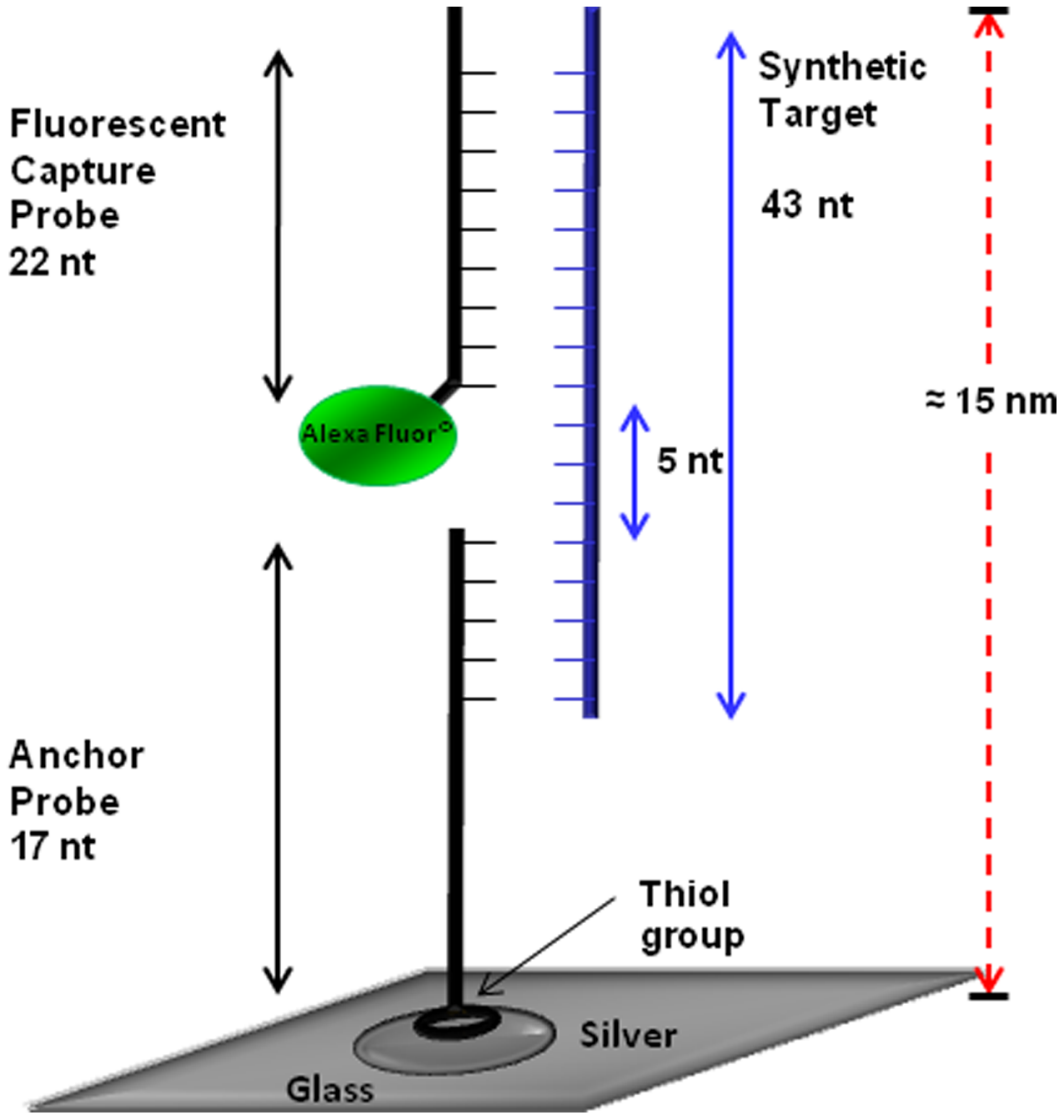
Schematic configuration of 3 piece DNA detection assay. This configuration was used for detection of both toxin A and toxin B probes. The anchor probe (17 oligonucleotides) was anchored to the SiFs by addition of the thiol group. The fluorescent probe (22 oligonucleotides) was attached to an Alexa at the 3′ end. Upon hybridization with target DNA, the 3 piece assay is formed, and the fluorophore labeled probe is plasmon enhanced.

Silver island Films (SiFs) were prepared at an OD_450_ of 0.4–0.5 on Silane-prep glass slides (Sigma Aldrich, USA; Figure 1; Figure 2) to enable silver adhesion, as described previously [33], [34].

### Anchor and fluorescent probes and target DNA

**Figure 3:**
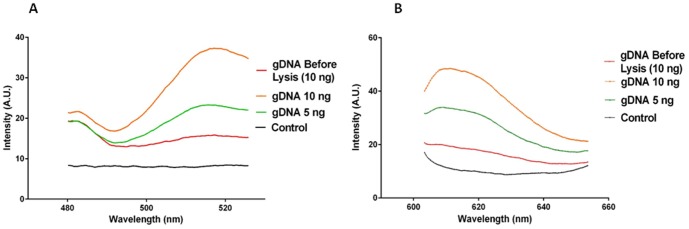
Detection of target DNA within various concentrations of gDNA by MAMEF. Various concentrations of *C. difficile* gDNA (strain CD630; ATCC, USA) were tested in the MAMEF platform. Concentrations of 10 ng and 5 ng were microwave irradiated for 8 seconds at 70% power. gDNA was subjected to disruption to increase the chances of the detection probes accessing the toxin specific sequences. The data presented is the result from a single reproduced assay. (A) The fluorescent signal intensities generated from toxin A detection within microwaved gDNA (excitation at 495 nm, emission at 519 nm). (B) The fluorescent signal intensities generated from toxin B detection within gDNA (excitation at 590 nm, emission at 617 nm). The control was PBS buffer.

**Figure 4:**
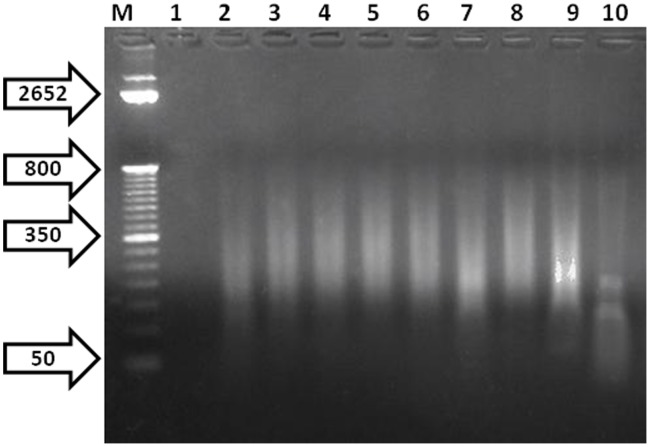
Electrophoresis gel of DNA released from *C. difficile* spores and vegetative cells following different periods of microwave treatment. *C. difficile* spores at a concentration of 1×10^5^ cfu/ml, in combination with gold triangles, were exposed to 80% microwave power for periods ranging from 8 to 16 seconds (Lanes 2–10). Lane M shows the 1 kilobase molecular weight DNA marker ladder. Lane 1 shows control non-microwaved bacteria.

Probes specific for conserved regions of toxin A and toxin B of *C. difficile* were designed and validated (Table 1; Figures 3, 4). Probes were modified to enable incorporation into the MAMEF detection platform (Figure 2); a thiol group was added to the 5′ region of the anchor probe to enable binding of the DNA to the surface of the SiF while the capture probe was labelled with an Alexa (Invitrogen, USA) fluorophore [20], [21]. The Alexa fluorophore 488 (green) was used to label toxin A and the Alexa fluorophore 594 (red) was used to label toxin B. These fluorophores were chosen due to their wavelength separation and thus yield two different fluorescence emissions upon excitation. The common regions in the anchor probes were preceded by 5 consecutive thymine bases, included to increase the flexibility of the probe once bound to the silver surface, and enhanced by the inclusion of a C at the 3′ terminal to which a thiol group was subsequently added [20]. The negative strand of the target region was also synthesised to bind to the capture and anchor probes as a synthetic control sample (Figure S1; Figure S2; Invitrogen, USA).

### Preparation of MAMEF assay platform for detection of *C. difficile* DNA

Glass slides with SiFs deposited were coated with self-adhesive silicone isolators as described previously [21]. The thiolated anchor probe was then covalently bound to the SiF in a self assembled monolayer chemistry [35]. This was achieved by diluting 40 µM anchor probe into 100 µl of 1 M Tris- EDTA buffer and adding 9 µl to 250 mM of 20 µl dithiothreitol. The mixture was incubated at room temperature for 60 min. The anchor probe (1 µM) was diluted into 4 ml Tris- EDTA buffer and 100 µl of the anchor probe was added to and incubated in each well of the SiF for 75 min. After incubation the anchor was removed and 50 µl of 1 µM Alexa-Fluor probe was added to 50 µl of DNA from the target organism. The SiFs containing the bound probes and DNA were then incubated by heating for 25 s in a microwave cavity at 20% power, GE microwave Model No. JE2160BF01, 1.65 kiloWatts. Only in the presence of target DNA the three piece assay is complete and enhanced-fluorescence can be observed (Figure 2). The sample was removed from the well after MAMEF and the well washed with 100 µl TE buffer 3 times. Each experiment was conducted in triplicate.

### Detection and fluorescence spectroscopy

The presence of target DNA was confirmed by the generation of a fluorescent signal following excitation with laser light. Fluorescence was emitted by the DNA MAMEF capture assay and measured using a Fiber Optic Spectrometer (HD2000) (Ocean Optics, Inc) by collecting the emission intensity (I).

### Statistical Analysis

Statistical analysis was performed using GraphPad Prism version 5.04 for Windows, (GraphPad Software, La Jolla California USA, www.graphpad.com). Three replicates were performed for each detection experiment. Statistical significant differences were tested for using one way analysis of variance (ANOVA) at the 95% confidence interval in conjunction with a Kruskall-Wallis test. Dunnett's post test was employed to compare sample data to control data. A P value of<0.05 was considered significant [36].

## Results

### Specificity of DNA probes for Toxin A and Toxin B detection as determined via dot blot

DNA probes were designed to recognize sequences within conserved regions of the gene sequences of toxin A and B and were configured to take account of the requirements of the MAMEF assay. Genomic DNA from 58 clinical isolates of *C. difficile*, representing a diverse range of ribotypes, was macroarrayed as shown in Figure S3. As expected each strain which contained a copy of the toxin A and B gene sequences gave a positive signal, the strength of which varied between isolates, likely due to an artifact of the experimental procedure. To confirm the specificity of the probes, variant isolates of *C. difficile* lacking toxin A (ribotypes 017; 047: *tcdA* ^−^ *tcdB* ^+^) or toxin B (Toxinotype XIa; XIb; DS1684: *tcdA* ^−^ *tcdB* ^−^) gene sequences were included in the panel. The DNA from each of these isolates was not recognized by the relevant probe.

To further confirm the specificity of the probes genomic DNA from other bacterial species, both close and distant relatives were subject to hybridization analysis (Figure S4; Table S2). The probes did not bind to bacterial species unrelated to *C. difficile*, further indicating that the probes were highly specific to toxins A and B of *C. difficile*. Species of the *Clostridium* genus, including species closely related to *C. difficile* (which possess toxins closely related to *tcdB*) did not show any probe hybridization which further validates probe specificity. We further confirm the specificity of these probes by demonstrating that they were unable to recognise metagenomic DNA from the human gut flora of ten healthy human volunteers (July 2011). Based on these results we concluded that the probes were highly specific and thus were suitable for inclusion in a MAMEF- based detection assay.

### Detection of toxin A and B sequences in genomic DNA isolated from *C. difficile* using the MAMEF assay

After confirming the specificity of the toxin A and B probes, their ability to bind to toxin gene sequences in the context of the MAMEF assay was assessed. gDNA isolated from a known toxin producing strain of *C. difficile* (CD630) was suspended in PBS buffer and subjected to microwave fragmentation for 8 seconds at 70% total microwave power [GE microwave Model No. JE2160BF01, kW 1.65 (M/W)]. DNA was subjected to disruption to increase the chances of the detection probes accessing the toxin specific sequences. Untreated gDNA in PBS was included as a control. Both sets of toxin specific probes were able to detect 10 ng of intact and disrupted gDNA (Figure 3A & B). The intensity of the signals generated against gDNA at emissions wavelengths of 519 nm (toxin A) and 617 nm (toxin B) were similar to those seen for synthetic oligonucleotide targets (Figures S1; S2) suggesting that microwave treatment is an efficient means of generating probe target DNA.

### Release of DNA from *C. difficile* spore preparations using focused microwave irradiation

Bacterial gDNA is normally sequestered within the body of the host organism, making it difficult for detection probes to gain access. The process is more complicated with bacteria such as *B. anthracis* and *C. difficile* which have the ability to form spores. Thus we employed a microwave based approach, previously developed to release DNA from *B. anthracis* spores, to break open the spore and vegetative form of *C. difficile* to release target DNA [20]. This approach employs gold triangles with a 1 mm gap to focus microwaves which rapidly heat the spores leading to their physical disruption. Thus 500 µl aliquots comprising 1×10^5^ cfu/ml of *C. difficile* spores suspended in PBS were subjected to microwave radiation for periods between 2 and 16 seconds, and then analyzed for DNA release via gel electrophoresis. Exposure to microwaves for 15 seconds at 80% total microwave power resulted in optimal release of DNA, with the majority of DNA disrupted to produce fragments between 50–200 bp in size (Figure 4). In contrast, irradiation for 16 seconds resulted in further fragmentation of DNA and yielding fragments too small to be detected by the toxin probes.

### Bacterial quantification following focused microwave irradiation

To confirm that microwave irradiation had resulted in disruption of bacterial structure and that the DNA detected did not simply represent free-floating extracellular DNA, the effect of microwave treatment on bacterial viability was determined. Exposure to microwaves for 15 seconds resulted in a 99% reduction in viable spore numbers from 1.33×10^7^ cfu/ml to 3.67×10^3^ cfu/ml.

### Detection of target DNA in spores of *C. difficile* suspended in PBS

**Figure 5:**
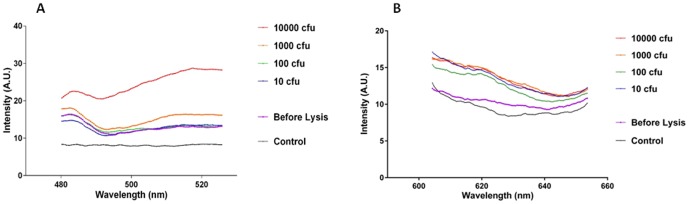
Detection of DNA from microwaved *C. difficile* spore preparation in PBS using MAMEF. Spores of *C. difficile* from strain CD630 at a range of concentrations 10, 100, 1000 and 10 000 cfu were suspended in PBS and subjected to microwave irradiation and screened for the presence of toxin A and toxin B using MAMEF. (A) Various fluorescent signal intensities generated from detection of toxin A within gDNA from irradiated spores (excitation at 495 nm, emission at 519 nm). (B) The various fluorescent signal intensities generated from detection of toxin B within gDNA from irradiated spores (excitation at 590 nm, emission at 617 nm).

**S**pores of *C. difficile* CD630 were suspended in PBS and microwaved for 15 seconds at 80% power. Following treatment, the cell suspension was diluted to give a range of bacterial concentrations (Figure 5A & B). At the highest spore concentration tested, 10000 cfu, the toxin A and B probes gave signals of 15 AU (519 nm) and 15 AU (617 nm) respectively. The lowest concentration of spores tested, 10 cfu, also gave detectable signals with both probes- (toxin A 12 AU, toxin B 10 AU).

### Detection of spores of *C. difficile* in human feces

**Figure 6:**
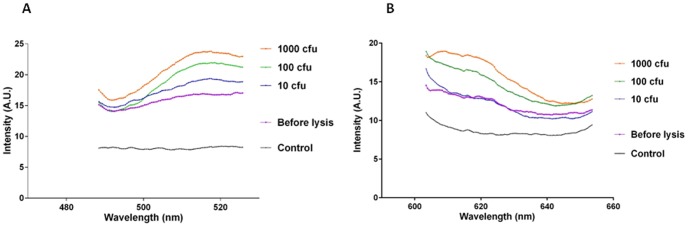
Detection of DNA from lysed *C. difficile* in feces using MAMEF. Spores of *C. difficile* from strain CD630 at a range of concentrations 10, 100, and 1000 cfu were suspended in human feces and subjected to microwave irradiation, then screened for the presence of toxin A and toxin B using MAMEF. (A) The various fluorescent signal intensities generated from detection of toxin A within gDNA from irradiated spores of *C. difficile* in human feces (excitation at 495 nm, emission at 519 nm). (B) Graph demonstrating the fluorescent signal intensities generated from detection of toxin B within gDNA from irradiated spores of *C. difficile* in human feces (excitation at 590 nm, emission at 617 nm).

Spores of *C. difficile* CD630 were suspended in human feces and microwaved for 15 seconds at 80% power. Prior to treatment, the spore suspension was diluted to give a range of bacterial concentrations (Figure 6A & B). At 1000 cfu, the toxin A and B probes gave signals of 23 AU (519 nm) and 19 AU (617 nm) respectively. The lowest concentration of spores tested, 10 cfu, also gave detectable signals with both probes- 15 AU (519 nm) and 13 AU (617 nm).

## Discussion

In this communication we describe the development of a bench top assay based on MAMEF which is capable of detecting as few as 10 bacteria of *C. difficile* in a fecal sample within 40 seconds. To put this into context, during infection patients excrete between 10 000 to 10 000 000 spores per gram of feces [37].

Thus, in addition to detecting active infection, the assay could also be used to identify patients who carry small numbers of *C. difficile* in their gut but are asymptomatic. The *C. difficile* asymptomatic carriage rate in humans varies between 4–20% and treatment of these individuals with broad spectrum antibiotics could precipitate an active infection [38]. Therefore information concerning the presence or absence of the bacterium in a patient's gut would assist the clinician in determining the most appropriate treatment option for that individual.

The level of sensitivity and speed of this prototype assay compares favorably with all of the currently available *C. difficile* detection methods [39]. The sensitivity of assays within the *C. difficile* detection market range from 90–100% for the cell cytotoxin, EIAs and PCR assays; however, to increase the sensitivity and accuracy of diagnosis these assays are employed in algorithms [40].

The ability of the assay to directly detect the pathogen in the presence of fecal material is a major advantage over other DNA based methods. Current PCR based assays can take between 2–8 hrs to generate a result; due in part to the need to remove enzyme inhibitors in feces which can prevent the amplification of target DNA [9]. In contrast, the MAMEF assay does not employ an enzymic amplification step and thus can operate in the presence of organic material such as blood and feces [22], [41]. This is due to the low power microwave acceleration which increases the rate of DNA hybridization within the biological sample via metal enhanced fluorescence; whereby the SiF amplifies the fluorescence of the labeled reporter DNA [42].

A further advantage of this prototype assay is its ability to detect the genes encoding both toxins A and B. The contribution of both toxins to the pathology of the infection is currently a subject of debate [8], [43]. Until relatively recently it was thought that only toxin B was essential for virulence. As a consequence commercial PCR based methods such as BD Gene Ohm and Xpert Cepheid are configured to detect only toxin B [44]. Given this uncertainty, the ability to detect both toxins would provide an obvious advantage until the relative importance of each toxin has been clarified.

To realize the potential of our promising technology, future studies will focus on optimizing the MAMEF platform. This will involve the development of an integrated microwave and laser platform miniaturization, reduced power signature and a simple readout would need to be incorporated into a hand held device capable of being used at the bedside. Studies are already in progress to multiplex the assay and thus detect the presence of both toxins A and B in a single sample well.

We believe our prototype *C. difficile* detection assay has the potential to be developed into a real-time assay capable of identifying infected and colonized individuals within 40 seconds. Such an assay would fulfill the pressing need for a rapid and sensitive detection system capable of detecting both toxin A and B. In the future we believe this assay could be used as a means of screening patients upon admission to hospital to enable appropriate treatment regimens and clinical management decisions to be made.

## Supporting Information

Figure S1 **Detection of various concentrations of synthetic oligonucleotide in TE buffer by MAMEF.** The concentration of anchor probe attached to the silver island film surface for toxin A was 10 nM, whereas for toxin B the anchor concentration was 100 nM. Thus there is variation in the signal intensity generated from the toxin A and toxin B probes. A range of synthetic oligonucleotide concentrations from 1 nM to 100 000 nM were used to determine the ability of the probes to fluoresce in response to excitation via laser light. The fluorescent intensity data presented in the above graphs is the result from a single reproduced assay (A) Graph demonstrating the various fluorescent signal intensities of a range of toxin A synthetic oligonucleotides bound to the toxin A anchor and detector probes. (B) Real color photographs of the fluorescent signal produced at each concentration from the sample wells. The laser light used to excite the fluorescent toxin A probe was at a wavelength of 495 nm excitation which produced an emission at 519 nm. (C) Graph demonstrating the various fluorescent signal intensities of a range of toxin B synthetic oligonucleotides bound to the toxin B anchor and detector probes. (D) Real color photographs of the fluorescent signal produced at each concentration from the sample wells. The laser light used to excite the fluorescent toxin B probe was at a wavelength of 590 nm excitation which produced an emission at 617 nm.
(TIF)

Figure S2 **Detection of various concentrations of synthetic oligonucleotides in feces by MAMEF.** Human feces was diluted by 50% in PBS and mixed with the synthetic target concentrations and tested in the MAMEF platform. The concentration of anchor probe attached to the silver island film surface for toxin A and toxin B was 10 nM. A range of synthetic oligonucleotide concentrations from 1 nM to 1000 nM were used to determine the ability of the probes to fluoresce in response to excitation via laser light. The fluorescent intensity data presented in the above graphs is the result from a single assay which was repeated three times. (A) Graph demonstrating the various fluorescent signal intensities of a range of toxin A synthetic oligonucleotides bound to the toxin A anchor and detector probes in the presence of human feces. (B) Graph demonstrating the various fluorescent signal intensities of a range of toxin B synthetic oligonucleotides bound to the toxin B anchor and detector probes in the presence of human feces.
(TIF)

Figure S3 **Dot blot of hybridization of** ***C. difficile*** **gDNA.** Genomic DNA from the panel of 58 *C. difficile* isolates tested were macroarrayed as shown (3A+B) and tested against our DIG-labeled probes. To confirm probe specificity, variant isolates of *C. difficile* (*tcdA* ^−^ *tcdB* ^+^) lacking either the toxin A (ribotypes 017; 047: *tcdA* ^−^ *tcdB* ^+^) or toxin B (Toxinotype XIa; XIb; DS1684: *tcdA* ^−^ *tcdB* ^−^) gene sequences were included. DNA from these isolates did not bind to the probes. (C) *tcdA*76 anchor probe (D) *tcdA*76 detector probe, (E) *tcdB* anchor probe (F) *tcdB* detector probe vs. *C. difficile* isolates. Bromophenol blue+ lambda phage DNA was added to the first well orientate the membrane.
(TIF)

Figure S4 **Dot blot of related and unrelated bacterial species by DNA hybridization.** Species related and unrelated to *C. difficile* were tested against the probes for specificity. There was no hybridization of the probes to the gDNA on the membrane. Positive control for each probe was CD630 and a variant strain control R22680 (*tcdA* ^−^ *tcdB* ^+^) was also included. (A) *tcdA*50 anchor probe, (B) *tcdA*50 detector probe, (C) *tcdA*76 anchor probe, (D) *tcdA*76 detector probe, (E) *tcdB* anchor probe, (F) *tcdB* detector probe.
(TIF)

Table S1 **Table of isolates used in this study.** The isolates of *C. difficile* are listed. Panel also includes 21 isolates as described previously [30]. Isolates from blood culture are listed as (B/C). Toxin production for each strain and its PCR ribotype are shown, with additional information including the source. The isolates and information above was provided courtesy of Dr. Jon Brazier and Dr. Val Hall at the Anaerobic Reference Unit, University Hospital Wales, Cardiff, UK, 2008.
(TIF)

Table S2 **Additional bacterial species used in this study.** The additional species and their strain designations used in this study are listed above. The species related to, and those not related to, *C. difficile* are shown. The species were obtained from the NTCC (National Type Culture Collection, HPA, London, UK), unless otherwise stated. Other isolates obtained from the anaerobic reference unit (ARU) at the University Hospital Wales (UHW) are listed as ARU, UHW. Those isolates and information was provided courtesy of Dr. Jon Brazier and Dr. Val Hall at the Anaerobic Reference Unit, University Hospital Wales, Cardiff, UK.
(JPG)

Table S3 **PCR Thermocycle annealing temperatures per probe.** The probes for each toxin were found to have the above optimal temperatures for PCR to occur. These are the temperature at which all further PCR reactions and dot blot reactions were conducted.
(TIF)
