# Supplementary figures and images for: Extraction and Sensitive Detection of Toxins A and B from the Human Pathogen Clostridium difficile in 40 Seconds Using Microwave-Accelerated Metal-Enhanced Fluorescence
Source: PLoS One. 2014 Aug 27;9(8):e104334. doi: 10.1371/journal.pone.0104334 (PMC4146460; doi:10.1371/journal.pone.0104334)

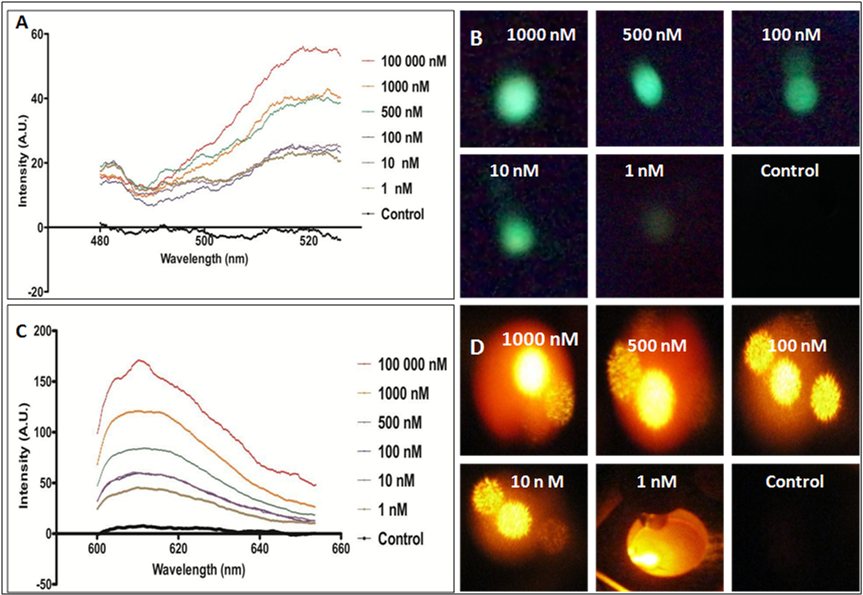

Supplement: Figure S1 — Detection of various concentrations of synthetic oligonucleotide in TE buffer by MAMEF. The concentration of anchor probe attached to the silver island film surface for toxin A was 10 nM, whereas for toxin B the anchor concentration was 100 nM. Thus there is variation in the signal intensity generated from the toxin A and toxin B probes. A range of synthetic oligonucleotide concentrations from 1 nM to 100 000 nM were used to determine the ability of the probes to fluoresce in response to excitation via laser light. The fluorescent intensity data presented in the above graphs is the result from a single reproduced assay (A) Graph demonstrating the various fluorescent signal intensities of a range of toxin A synthetic oligonucleotides bound to the toxin A anchor and detector probes. (B) Real color photographs of the fluorescent signal produced at each concentration from the sample wells. The laser light used to excite the fluorescent toxin A probe was at a wavelength of 495 nm excitation which produced an emission at 519 nm. (C) Graph demonstrating the various fluorescent signal intensities of a range of toxin B synthetic oligonucleotides bound to the toxin B anchor and detector probes. (D) Real color photographs of the fluorescent signal produced at each concentration from the sample wells. The laser light used to excite the fluorescent toxin B probe was at a wavelength of 590 nm excitation which produced an emission at 617 nm. (TIF) [file pone.0104334.s001.tif]

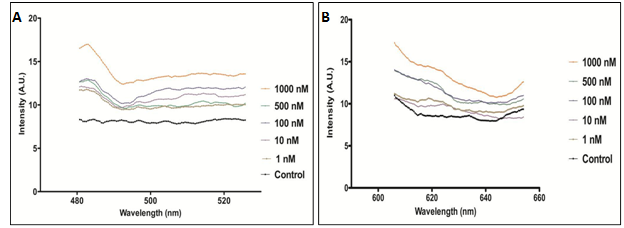

Supplement: Figure S2 — Detection of various concentrations of synthetic oligonucleotides in feces by MAMEF. Human feces was diluted by 50% in PBS and mixed with the synthetic target concentrations and tested in the MAMEF platform. The concentration of anchor probe attached to the silver island film surface for toxin A and toxin B was 10 nM. A range of synthetic oligonucleotide concentrations from 1 nM to 1000 nM were used to determine the ability of the probes to fluoresce in response to excitation via laser light. The fluorescent intensity data presented in the above graphs is the result from a single assay which was repeated three times. (A) Graph demonstrating the various fluorescent signal intensities of a range of toxin A synthetic oligonucleotides bound to the toxin A anchor and detector probes in the presence of human feces. (B) Graph demonstrating the various fluorescent signal intensities of a range of toxin B synthetic oligonucleotides bound to the toxin B anchor and detector probes in the presence of human feces. (TIF) [file pone.0104334.s002.tif]

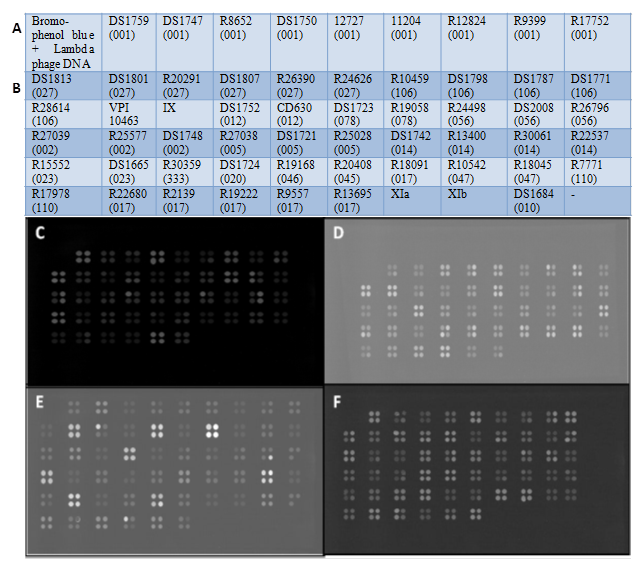

Supplement: Figure S3 — Dot blot of hybridization of C. difficile gDNA. Genomic DNA from the panel of 58 C. difficile isolates tested were macroarrayed as shown (3A+B) and tested against our DIG-labeled probes. To confirm probe specificity, variant isolates of C. difficile (tcdA − tcdB +) lacking either the toxin A (ribotypes 017; 047: tcdA − tcdB +) or toxin B (Toxinotype XIa; XIb; DS1684: tcdA − tcdB −) gene sequences were included. DNA from these isolates did not bind to the probes. (C) tcdA76 anchor probe (D) tcdA76 detector probe, (E) tcdB anchor probe (F) tcdB detector probe vs. C. difficile isolates. Bromophenol blue+ lambda phage DNA was added to the first well orientate the membrane. (TIF) [file pone.0104334.s003.tif]

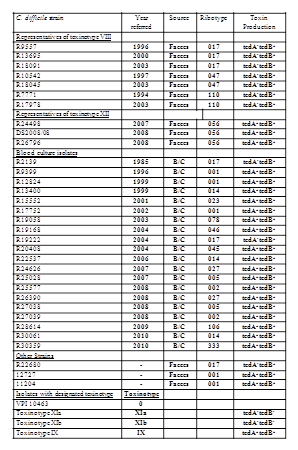

Supplement: Table S1 — Table of isolates used in this study. The isolates of C. difficile are listed. Panel also includes 21 isolates as described previously [30]. Isolates from blood culture are listed as (B/C). Toxin production for each strain and its PCR ribotype are shown, with additional information including the source. The isolates and information above was provided courtesy of Dr. Jon Brazier and Dr. Val Hall at the Anaerobic Reference Unit, University Hospital Wales, Cardiff, UK, 2008. (TIF) [file pone.0104334.s005.tif]

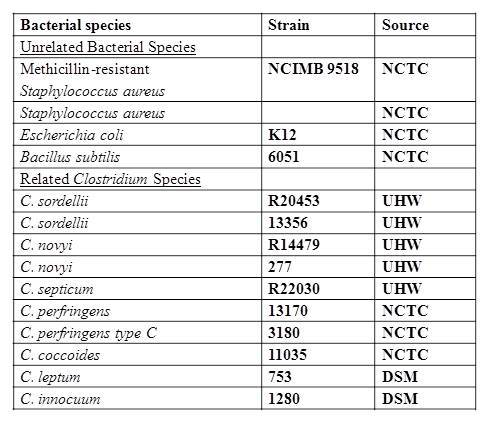

Supplement: Table S2 — Additional bacterial species used in this study. The additional species and their strain designations used in this study are listed above. The species related to, and those not related to, C. difficile are shown. The species were obtained from the NTCC (National Type Culture Collection, HPA, London, UK), unless otherwise stated. Other isolates obtained from the anaerobic reference unit (ARU) at the University Hospital Wales (UHW) are listed as ARU, UHW. Those isolates and information was provided courtesy of Dr. Jon Brazier and Dr. Val Hall at the Anaerobic Reference Unit, University Hospital Wales, Cardiff, UK. (JPG) [file pone.0104334.s006.jpg]

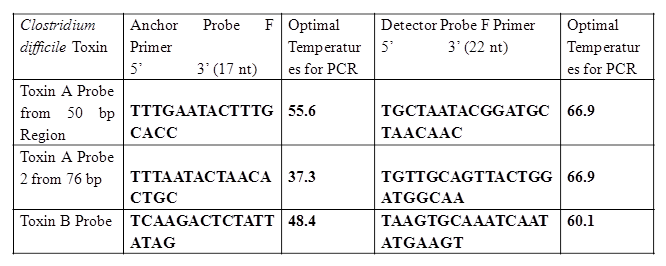

Supplement: Table S3 — PCR Thermocycle annealing temperatures per probe. The probes for each toxin were found to have the above optimal temperatures for PCR to occur. These are the temperature at which all further PCR reactions and dot blot reactions were conducted. (TIF) [file pone.0104334.s007.tif]
